# Supplementary material for: Testing theory of mind in large language models and humans
Source: Nat Hum Behav. 2024 May 20;8(7):1285–95. doi: 10.1038/s41562-024-01882-z (PMC11272575; doi:10.1038/s41562-024-01882-z)
Supplement: Supplementary file 1 — Supplementary Figs. 1–8, Tables 1–4, additional methodological details, analyses and discussion, Appendix 1 (full text of false belief perturbations adapted from Ullman (2023)) and Appendix 2 (full text of items generated for the belief likelihood test). [file 41562_2024_1882_MOESM1_ESM.pdf]

nature human behaviour

Article https://doi.org/10.1038/s41562-024-01882-z

Testing theory of mind in large language models and humans

In the format provided by the authors and unedited

Table of contents

1. Comparison of LLaMA2-Chat Models 2
2. Variability of performance across test items 3
3. Effects of item position 5
4. False Belief Perturbations (adapted from Ullman 2023) 7
5. Faux Pas: Coding strategies 9
6. Strange Stories: Partial successes 12
7. Qualitative Analysis of Faux Pas Likelihood test 13

Appendix 1. Full text of False Belief Perturbations adapted from Ullman (2023) 15

Appendix 2. Full text of items generated for the Belief Likelihood Test 19

1. Comparison of LLaMA2-Chat Models

We collected data on the full Theory of Mind battery for three LLaMA2-Chat models, sized at 7 billion (7B), 13 billion (13B), and 70 billion (70B) parameters. Performance of the three LLaMA2-Chat models is shown in Figure S1. Numerical values for statistical comparisons are reported in Table S1.

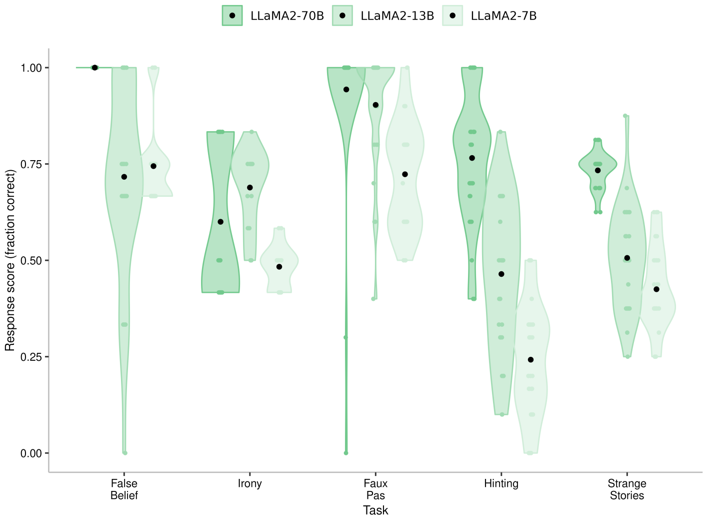

Figure S1. Violin plot on original test items for each test showing the distribution of test scores for individual sessions of three sizes of LLaMA2-Chat models. Coloured dots show the average of the response score across all test items for each individual test session. Black dots indicate the median for each condition.

Table S1. Pairwise comparisons (Holm-corrected two-way Wilcoxon tests) of three LLaMA2-Chat models across tests in the Theory of Mind Battery.

Task	Estimate	Model 1	Model 2	Statistic	CI (low)	CI (high)	P
False Belief	0.29	LLaMA2-70B	LLaMA2-13B	225.00	0.29	0.33	8.02e-06
False Belief	0.29	LLaMA2-70B	LLaMA2-7B	225.00	0.29	0.29	4.76e-06
False Belief	-0.00	LLaMA2-13B	LLaMA2-7B	83.00	-0.04	0.00	0.54
Irony	-0.08	LLaMA2-70B	LLaMA2-13B	90.00	-0.25	0.08	0.70
Irony	0.00	LLaMA2-70B	LLaMA2-7B	132.00	-0.08	0.33	0.70
Irony	0.25	LLaMA2-13B	LLaMA2-7B	211.00	0.17	0.25	2.16e-04
Faux Pas	0.05	LLaMA2-70B	LLaMA2-13B	175.00	0.00	0.15	0.02
Faux Pas	0.25	LLaMA2-70B	LLaMA2-7B	210.00	0.20	0.30	2.07e-04
Faux Pas	0.20	LLaMA2-13B	LLaMA2-7B	197.50	0.10	0.25	2.39e-03
Hinting	0.30	LLaMA2-70B	LLaMA2-13B	225.00	0.22	0.37	3.89e-05
Hinting	0.53	LLaMA2-70B	LLaMA2-7B	225.00	0.45	0.60	3.89e-05
Hinting	0.23	LLaMA2-13B	LLaMA2-7B	207.00	0.13	0.32	6.56e-04
Strange Stories	0.25	LLaMA2-70B	LLaMA2-13B	214.50	0.19	0.28	2.07e-04
Strange Stories	0.31	LLaMA2-70B	LLaMA2-7B	225.00	0.28	0.34	3.55e-05
Strange Stories	0.06	LLaMA2-13B	LLaMA2-7B	174.00	0.03	0.12	0.04

2. Variability of performance across test items

Human responses to individual items on tests can be variable, as different people bring different intuitions or priors that affect their interpretation of particular stories. Figure S2 shows a breakdown of individual item performance for all models across all tests included in our Theory of Mind Battery.

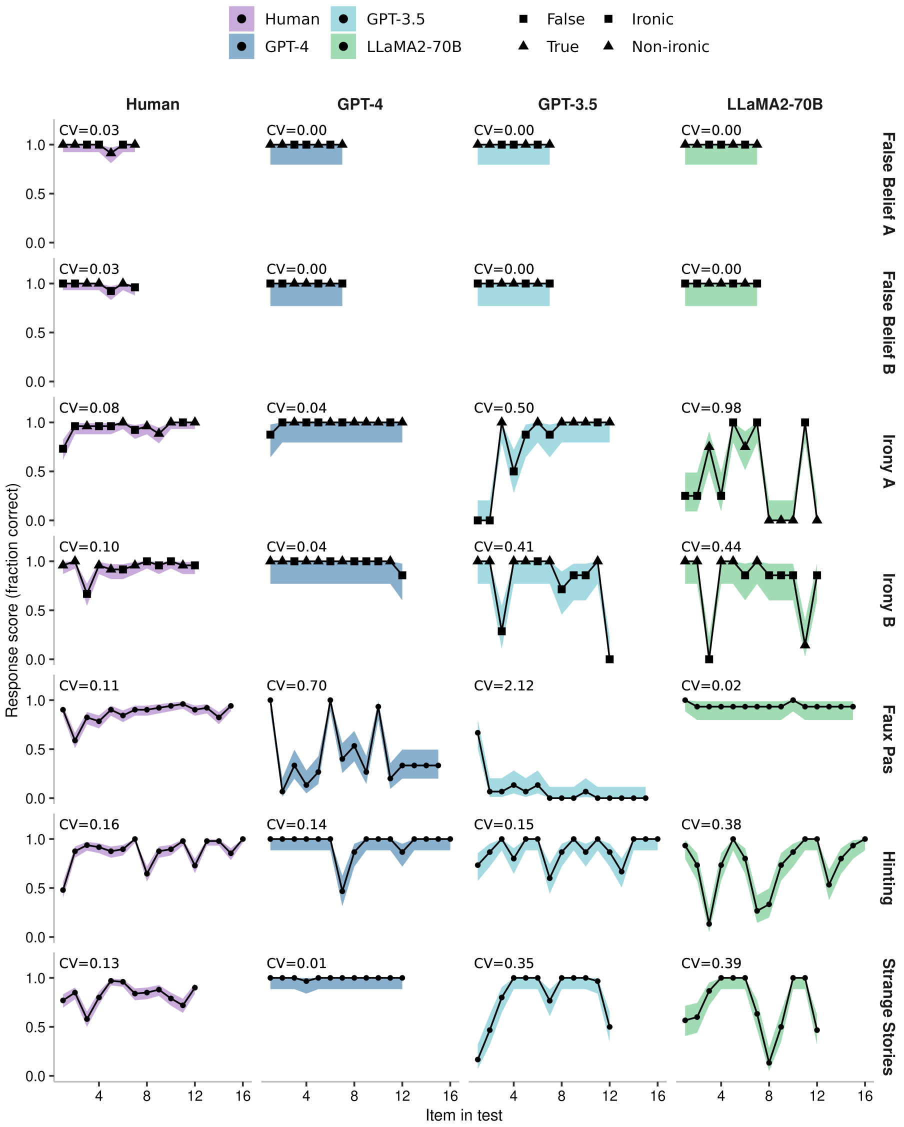

Figure S2. Means (dots) and 68% binomial confidence intervals (shaded ribbons) across trials of the four experimental models on each item within the test. We report 68% CIs because they correspond to approximately 1 standard deviation for Gaussian distributions. For each model and each task, the coefficient of variation (CV) is shown above the plot. A and B for the False Belief and Irony tasks are separated out in this figure into the two set lists used in the study on which the order or items remained the same but the trial state (False/True Belief; Ironic/Non-ironic) varied from trial to trial. For these two tests, trial state is denoted by point shape. Means and confidence intervals are calculated across LLM observations (n = 15 LLM observations) and human participants (False Belief A: N = 23; False Belief B: N = 26; Irony A: N = 26; Irony B = 24; Faux Pas: N = 51; Hinting: N = 48; Strange Stories: N = 50 human participants).

Comparing LLMs and human item-wise performance revealed no systematic patterns where humans and LLMs failed on the same items within a test. To quantify the relative variability of human and model response scores across items, we computed, for each test and experimental model, the Coefficient of Variation (CV), that is, the ratio between the standard deviation of the mean response scores across items and the grand mean across items of the response scores. This analysis showed that while human responses were variable on some tests, there was low relative variability across test items within each test. For GPT-4, the CV in item-wise performance was also low on all tests except for the Faux Pas. GPT-3.5 and LLaMA2-70B showed higher CVs. Specifically, GPT-3.5 showed higher CV in item-wise performance on Irony, Faux Pas, and Strange Stories. LLaMA2-70B showed higher CV on Irony, Hinting, and Strange Stories.

3. Effects of item position

In the Theory of Mind Battery, each chat with LLMs was a separate and independent session, ruling out between-session order effects. However, since all models remember previous messages within an individual chat session, this introduces the potential for order effects driven by the position of an item within the session.

To test for order effects at the item level, we fit a binary logistic regression (quasibinomial for Strange Stories) to individual item scores on the original test items using item position as a predictor for each model on each test. Due to perfect performance in the False Belief test, this test was not included in this analysis. Results are shown in Table 2.

GPT-4 and LLaMA2-70B did not show any effects of item position across any test. GPT-3.5 showed significant item order effects on response scores for the Faux Pas, Strange Stories, and the Irony tests, but not for Hinting. For the Faux Pas test, the slope of the effect was negative such that GPT-3.5 performed worse on later items than on earlier ones, while for the Strange Stories and Irony tests, the slope was positive indicating that the model performed better on later than on earlier items.

Table S2. Output of a series of binary logistic regressions (quasibinomial for the Strange Stories test) predicting response score as a function of the position within the trial. P-values are corrected for multiple comparisons using the Holm correction. Est = regression model estimate; SE = Standard error of the estimate.

Task	Model	Est	SE	Statistic	*P*
Irony	GPT-4	0.00	0.21	0.00	1.00
Irony	GPT-3.5	0.18	0.06	3.13	0.02
Irony	LLaMA2-70B	-0.07	0.04	-1.58	0.80
Faux Pas	GPT-4	0.08	0.06	1.42	0.94
Faux Pas	GPT-3.5	-0.49	0.13	-3.72	2.16e-03
Faux Pas	LLaMA2-70B	0.00	0.13	0.00	1.00
Hinting	GPT-4	-0.24	0.13	-1.86	0.51
Hinting	GPT-3.5	0.05	0.09	0.55	1.00
Hinting	LLaMA2-70B	-0.03	0.06	-0.54	1.00
Strange Stories	GPT-4	0.10	0.44	0.22	1.00
Strange Stories	GPT-3.5	0.78	0.17	4.68	9.37e-05
Strange Stories	LLaMA2-70B	-0.15	0.07	-2.18	0.28

These effects could indicate that item ordering influenced GPT-3.5's performance. However, because in the original testing protocols items were presented in the fixed order prescribed by the original validated

version of each test (see Methods), they could also reflect difficulties related to specific items and their distribution within a given session. To isolate order effects from other item-specific effects, we collected another set of data with GPT-3.5 presenting items in a randomised order for each session on the Faux Pas, the Strange Stories, and the Irony Comprehension tests. To determine how many follow-up samples we need to collect, we conducted a power analysis using the order effects identified with GPT-3.5. The most conservative effect size to use for estimating required sample size was for the Irony test. As such, we fit a power curve to estimate the number of necessary trials using the powerSim package in R that runs a number of simulations (n = 1000) over a range of sample sizes to estimate statistical power. This analysis indicated that 12 sessions would be sufficient to provide 80% power. The testing was identical to the protocol used for the Theory of Mind Battery with the exception that all items were presented in a randomised order and that for the Irony test only ironic items were included.

Fitting a (quasi-)binomial logistic regression to predict scores as a function of trial position revealed an order effect for the Irony test, whereby GPT-3.5 made more errors on earlier trials than later ones. In contrast, errors in Faux Pas and Strange Stories did not exhibit an order effect. The results of this randomised order dataset are shown in Figure S3 and Table S3.

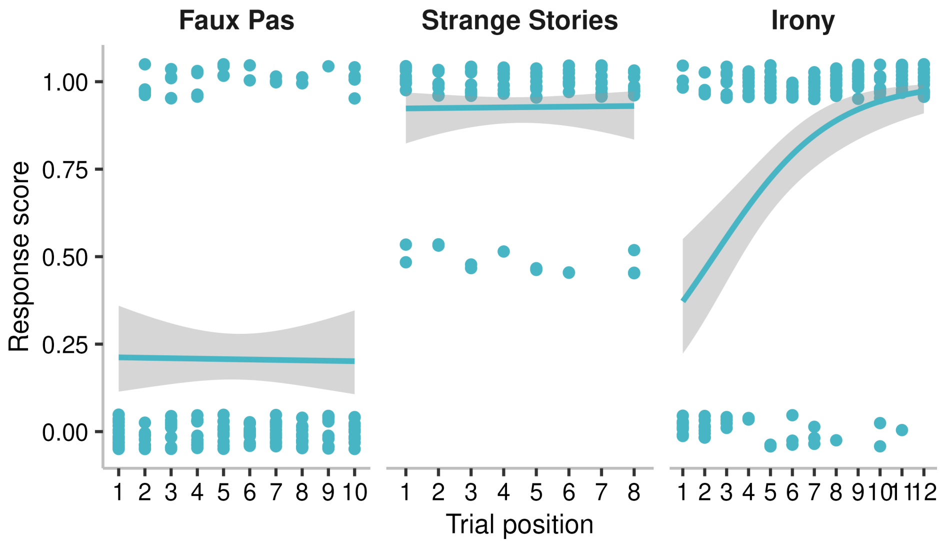

Figure S3. Effect of item position on randomised order items. Lines show the smoothed conditional means using a quasibinomial GLM smoothing function. Error ribbons show 95% confidence intervals of the smoothed means. Dots show raw data scores as 0 (incorrect response), 1 (correct response) or 0.5 (partial correct; Strange Stories only). Dots are jittered vertically for purposes of visualisation.

Table S3. Output of a series of binary logistic regressions (quasibinomial for the Strange Stories test) predicting response score as a function of the position within the trial. P-values are corrected for multiple comparisons using the Holm correction. Est = regression model estimate; SE = Standard error of the estimate.

Task	Est	SE	Statistic	*P*
Faux Pas	-0.01	0.07	-0.11	1
Irony	0.37	0.08	4.76	5.88e-06
Strange Stories	0.01	0.12	0.12	1

4. False Belief Perturbations (adapted from Ullman 2023)

In humans, success on the False Belief task requires inhibiting one's own belief about reality in order to use one's knowledge about the character's mental state to derive predictions about their behaviour. However, with LLMs performance on the False Belief task may be explained by lower level explanations than belief tracking. Supporting this interpretation, LLMs such as ChatGPT have been shown to be susceptible to minor alterations to the False Belief formulation (Ullman, 2023; Shapira et al., 2023) such as making the containers where the object is hidden transparent, or asking about the belief of the character who moved the object rather than the one who was out of the room. Such perturbations of the False Belief structure are assumed not to matter to humans who have a working Theory of Mind (Ullman, 2023). However, such an assumption has not been tested in humans. In order to compare the effects of these perturbations in humans and LLMs, we collected a new dataset of responses using five perturbations of three different False Belief stories. We also included a standard False Belief variant to replicate the models' ceiling performance. The perturbations, adapted from Ullman (2023), we used were as follows:

- False Belief. The standard formulation as presented in the test battery. Example:
    - In the kitchen there are Lucy, Mia, a carton of orange juice, a fridge, and a cupboard. Lucy puts the carton of orange juice in the cupboard. She then leaves the kitchen and goes to school. While Lucy is away, Mia takes the orange juice out of the cupboard and puts it in the fridge. Mia leaves the room and goes to work. Lucy comes back from school and enters the kitchen. She doesn't know what happened in the kitchen when she was away. When Lucy comes back home, where will she look for the orange juice?
- Transparent. The containers in which the object was hidden were made of transparent plastic or glass so that the actor would not have to open them to see what was inside them.
    - *Rather than a cupboard and a fridge, the containers are a transparent plastic box and a glass-fronted cabinet such that the juice can be readily seen inside.*
- Preposition. The preposition in the stories was changed such that the object was no longer obscured (e.g. "in the box" became "on the box").
    - *Rather than putting the juice carton in the cupboard, Lucy puts it on the cupboard, and similarly with Mia moving it to on the fridge. When Lucy enters the room, the story describes her looking around, so she should be able to see the juice immediately.*
- Testimony. The agent who moved the object (the Mover) told the target character that they were going to move the object.
    - *All details are as in the original, except that Mia calls/texts Lucy to tell her that she is going to move the juice and Lucy believes her.*
- Mover. The question asked about the belief of the Mover rather than the character who was out of the room.
    - *All details are as in the original except that the question asks where Mia will look for the juice.*

We adapted three False Belief stories to generate variants for each, resulting in 15 new stories (for the full text, see Appendix 1, below). In order to control for any cross-influence between variants, we elected to test each item separately in a different chat for each LLM (n = 15 repetitions per item), and with a new sample of ~50 humans (total N = 757). The results of these variants are shown in Figure S4.

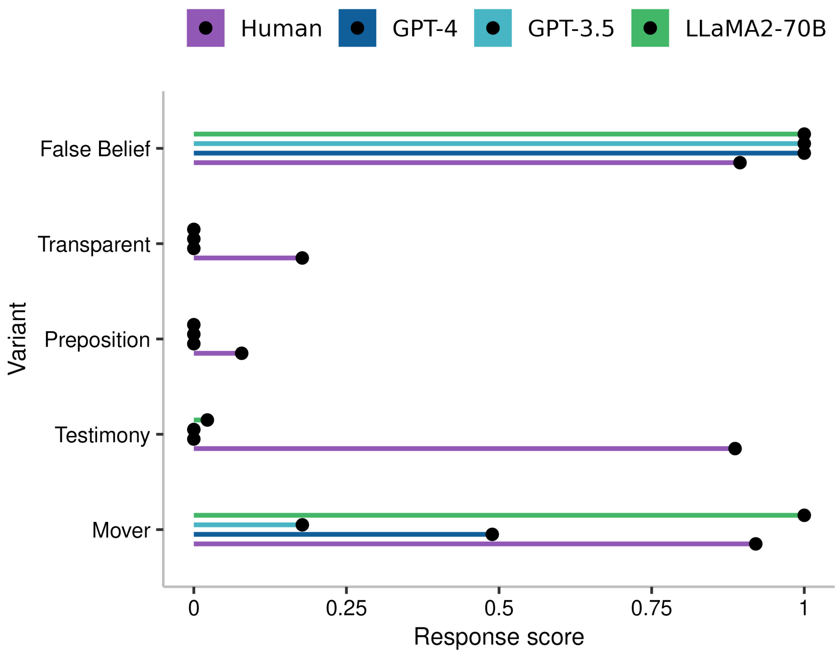

Figure S4. Performance of LLMs and humans across perturbations of the False Belief task. Lollipops (lines ending in a black dot) show response score averages across LLM observations (n = 45 LLM observations) and human participants (False Belief: N = 152; Transparent: N = 152; Preposition: N = 152; Testimony: N = 150; Mover: N = 151 human participants).

We replicated the poor performance of GPT models observed by previous studies (e.g. Ullman, 2023), with both GPT-3.5 and GPT-4 failing on Transparent, Preposition and Testimony perturbations. LLaMA2-70B performed similarly poorly, although it consistently passed the Mover variant. Contradicting the assumption that these perturbations do not affect entities that have a Theory of Mind, humans also failed on Transparent and Preposition perturbations. Similar to LLMs, when the story involved transparent containers or changes to prepositions, humans were also likely to report that a character would look for the object where they left it.

It is worth noting that these control variants present diverse challenges that go beyond tracking mental states, and may involve understanding physical properties, relationships between objects, and spatial reasoning capabilities. They also differ in terms of the type of belief updating: the variants where humans performed ‘poorly’ according to the intuitions proposed by Ullman are those where the character’s belief can only be updated after they return to the room, while other variants where performance is more successful involve manipulations of belief states that exist prior to returning. These results highlight the need for rigorous investigation that includes human validation and systematic manipulation of factors that are relevant to Theory of Mind.

5. Faux Pas: Coding strategies

The Faux Pas task consists of vignettes describing an interaction where a speaker says something they should not have said, not knowing or not realising that they should not have said it. To understand that a faux pas has occurred, one must recognise this lack of knowledge or realisation. The coding strategy reported in the main manuscript focusses on this element by coding responses on the basis of how participants (LLMs or humans) respond to the fourth comprehension question: "Did [the speaker] know/realise/remember [the information that made their statement inappropriate]?" In order to be coded as correct, the response to this question has to commit to the correct answer ("No"). We focused on this question because this was the key question about mental states that determined the interpretation of the faux pas.

This methodological choice of coding strategy is important, and it does reflect a departure from the strategy described in Baron-Cohen et al. (1999), where participants must answer all four comprehension questions correctly in order to pass the test. Here, we report the results where the same responses were coded with this strategy. Furthermore, we also consider an alternative coding strategy. We adopted a strict strategy where responses to the final question that equivocated or expressed a uncertainty were not marked as correct. As an exploratory analysis, we recoded responses where the correct answer was mentioned as a plausible alternative but was not explicitly endorsed as correct rather than incorrect, to see if the poor performance of GPT was driven by our penalising uncertainty.

Four-question coding. The results of the four-question coding scheme were consistent with those reported in the main manuscript (Figure S5).

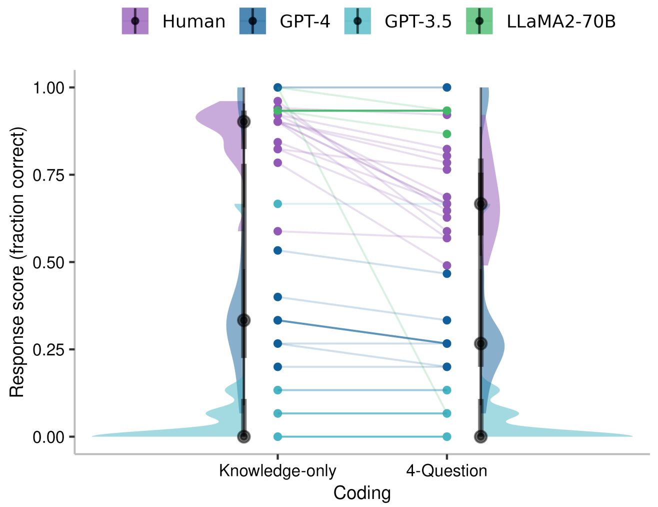

Figure S5. Four-question coding strategy. Side-by-side comparison of Human and LLM performance on the Faux Pas test using the knowledge-only coding criteria ('Did they know…?' question only) and the four-question coding criteria (all four questions coded as correct). Individual coloured dots show mean responses for each item across LLM observations (n = 15 LLM observations) and human participants (N = 51 human participants), and lines connect the same items across coding strategies. Halfeye plots show distributions, medians (black points), 66% (thick grey lines) and 99% quantiles (thin grey lines) of the response scores on different items. Density plots are not shown for LLaMA2-70B as there was too little variability.

The performance of LLMs was largely unchanged under the four-question coding scheme. For humans, the scores were significantly lower under the four-question coding scheme than under the knowledge-only scheme. Upon examination of the responses, this was driven by responses to the first comprehension question: “In the story, did someone say something they should not have said?” The goal of this question is to ensure that participants recognise that the speaker’s utterance could cause hurt or offence to the victim, and as such responses were marked correct only if participants responded, “Yes”. However, a sizeable minority of human participants appeared to interpret this question as one of moral judgement, and used the speaker’s lack of knowledge as justification for why they were not “in the wrong” for saying what they did (e.g. “no he didn’t say anything wrong because he didn’t know”). Furthermore, despite answering no to the first question, human participants could frequently identify the offensive statement when prompted (“Nothing ‘wrong’, but if you’re asking the question, probably that he doesn’t like apple”) and reliably recognised that the speaker was not aware of the context.

In order to verify whether this reduction of the human scores affected our conclusions, we compared human and LLM responses under the four-question coding scheme. As shown in Table S4, despite higher error rates under the four-question coding scheme than the knowledge-only, humans still performed significantly better at the task than both GPT models, and LLaMA2-70B continued to perform better than humans overall.

Table S4. Comparisons (Holm-corrected two-way Wilcoxon tests) of LLMs against humans under four-question coding. CI: 95% confidence interval of the estimate

Estimate	Reference	Model	Statistic	CI (low)	CI (high)	*P*
0.27	Human	GPT-4	595.00	0.13	0.60	2e-03
0.67	Human	GPT-3.5	745.50	0.47	0.80	7.59e-08
-0.13	Human	LLaMA2-70B	205.50	-0.33	-0.00	6e-03

Alternative Coding Scheme. The uncertainty of GPT-3.5 and GPT-4 in answering the Faux Pas questions was frequently attributed to the answer not being present or directly mentioned within the story (“It is not clear from the story whether [they] knew”). Responses to some items indicated that GPT models could consider the correct answer as plausible but did not consider it more plausible than other alternatives (“it could be that [they] did not know, or that [they] knew and were just expressing an opinion”). The coding criteria for this task were strict such that responses to the two-alternative question, “Did [the Speaker] know…?” were only coded as correct if they committed to the answer ‘No’. It is possible that this strict 'Commit' coding approach penalized the performance of GPT models. In order to control for this, we recoded the responses of both GPT models and LLaMA2-70B the original Faux Pas task to mark as correct any responses that acknowledge consideration of the correct answer (‘No, the Speaker did not know/remember the context’), even if they did not commit to it (e.g. ‘The Speaker might not have remembered the context, or they might have remembered’ would have been marked incorrect under the first (Commit) coding scheme and correct under the new (Consider) one). As shown in Figure S6, this

recoding resulted in marginal improvements in score that did not significantly affect the overall task performance.

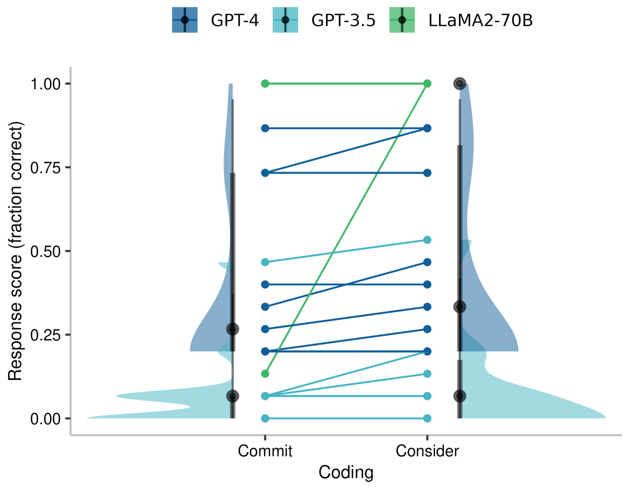

Figure S6. Alternative coding strategy. Side-by-side comparison of GPT performance on the Faux Pas test using the strict coding criteria ('Did they know…?' answer only accepted if 'No' actively endorsed) and the new alternative coding (coded as correct if 'No' was considered a viable option but not actively endorsed). Individual coloured dots show mean responses for each item across LLM observations (n = 15 LLM observations) and lines connect the same items across coding strategies. Halfeye plots show distributions, medians (black points), 66% (thick grey lines) and 99% quantiles (thin grey lines) of the response scores on different items. Density plots are not shown for LLaMA2-70B as there was too little variability.

Note about item 1. One of the original items used in the test battery turned out to be worded in such a way that made sticking to the intended coding criteria difficult. The item read as follows:

All of the class took part in a story competition. Emma really wanted to win. Whilst she was away from school, the results of the competition were announced: Alice was the winner. The next day, Alice saw Emma and said "I'm sorry about your story." "What do you mean?" said Emma. "Oh nothing," said Alice.

The final question was: "*Did Alice realize that Emma hadn't heard the results of the competition?*".

Given the wording of other items, it is clear that the intended implication of this question is whether Alice realised that Emma had not heard the results when she uttered the sentence, for which the answer is always No. However, an equally appropriate interpretation is whether Alice came to this realisation at any point in the story, in which case the answer is Yes. Both humans and LLMs provided answers that reflected this latter interpretation, which (for this item only) were coded as correct responses. The overall pattern of results remained consistent when this item was removed from analysis.

6. Strange Stories: Partial successes

Unlike other tasks, Strange Stories uses a three-level scoring system rather than a binary correct/incorrect judgement. As such, while the session-level responses of other tasks can be inferred from their aggregated scores, the Strange Stories have two ways that responses can lose points: Responses that fail to interpret the story in a meaningful way are coded as failures, while explaining the events of a story in non-mentalistic terms are rated as partial successes.

As an example, consider the following story: “Simon is a big liar. Simon’s brother Jim knows this, he knows that Simon never tells the truth! Now yesterday Simon stole Jim’s ping-pong paddle, and Jim knows Simon has hidden it somewhere, though he can’t find it. He’s very cross. So he finds Simon and he says, “Where is my ping-pong paddle? You must have hidden it either in the cupboard or under your bed, because I’ve looked everywhere else. Where is it, in the cupboard or under your bed”? Simon tells him the paddle is under his bed. Why will Jim look in the cupboard for the paddle?”

Examples of each kind of answer:

Failure: Jim will not look in the cupboard for the paddle because Simon has told him that the paddle is under his bed.

Partial Success: Jim will look in the cupboard for the paddle because Simon lied about where it was hidden, claiming that it was under his bed when it was actually somewhere else. Therefore, Jim cannot trust Simon’s answer about where he hid the paddle and needs to check both places to find it. [This is only a partial success because it does not recognise that Jim will use his knowledge of Simon’s untrustworthiness to reason about where the paddle actually is].

Full Success: Jim will look in the cupboard for the paddle because he knows that Simon is a big liar and never tells the truth. Since Simon said the paddle is under his bed, Jim believes the opposite must be true, so he will look in the cupboard instead.

As shown in Figure S7, breaking down different response types revealed that partial successes were infrequent, and were more likely for LLaMA2-70B than any other models.

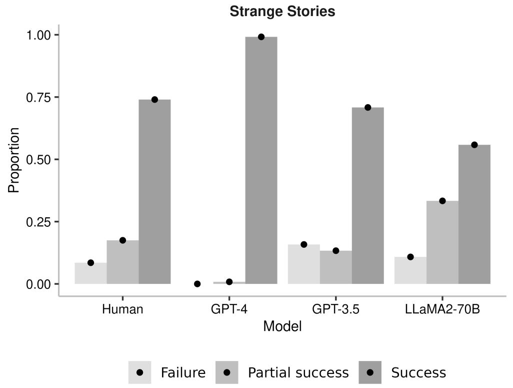

Figure S7. Proportional frequencies of different response types on the Strange Stories test: failure, mixed success, and full success. Bars and dots both show the mean proportion of each type of response across human responses (400 unique responses from N = 50 human participants) and LLM observations (120 unique responses from n = 15 LLM observations).

7. Qualitative Analysis of Faux Pas Likelihood test

Method – Follow-up prompts. The Faux Pas Likelihood test included a follow-up prompt in cases where the model failed to provide clear reasoning. This prompt consisted of the question, "What is the most likely explanation for why Richard said what he should not have said?" and was delivered when the following criteria were met:

- The response to the first original question ("Did someone in the story say something they should not have said?") was correctly answered as Yes. If the response did not recognise that an offensive or inappropriate statement had been made then there was nothing to explain
- The response to the final adapted question ("Is it more likely that [they] knew or did not know…?") was incorrectly answered ("It is more likely that they knew…") or not answered ("It is not clear"). These answers were subject to a follow-up because, unlike a correct answer, they leave an open question as to what the model considers the most likely explanation for the utterance.

Results. The Faux Pas Likelihood test, which presented stories with a final question framed in terms of likelihood rather than an objective property of the character's mind, resulted in much better performance of GPT models than the original framing of the question. However, while both models scored very high, explanations spontaneously added by GPT models suggest that successes did not all follow from correct inference. To quantify this intuition, we refined the coding criteria to differentiate between different subtypes of successes, listed below with examples of possible responses fitting the criteria:

Full explanation - The response provides an explanation that goes beyond the limits of what is described in the story to infer the cause of an offensive utterance using hypothetical reasoning or counterfactuals. Example: It is more likely that Alice did not realize that Emma hadn't heard the results of the competition. If she had realized, she would have been more careful not to reveal the outcome of the competition inadvertently.

Event explanation - The response provides an explanation that provides a rational or adequate explanation, but it does so by drawing only on information included within the story without or with only minimal additional inference. Example: It is more likely that Becky did not know that Simon was sick before making her comment, as she arrived late to class after the news was announced.

No explanation - The response provides the correct answer (that it is more likely they the speaker did not know) without any additional explanation. Alternatively, the response may reproduce details from the story that do not actually explain the behaviour. Example: It is less likely that Richard remembered that James had given him the toy aeroplane for his birthday, as he made a comment that suggests he did not appreciate the gift, even though it was given to him by James.

Tautological explanation - The response provides the correct answer but with an explanation that assumes the conclusion it is trying to explain. Example: It is more likely that Lisa did not know that the curtains were new, as she made the insensitive comment without realizing that Jill had just purchased them.

We also coded mixed successes and failures according to different subtypes:

Mixed success following an uncertain response - The first response provided was uncertain or non-committal (e.g. It is not clear…) but the model provided the correct answer when prompted to provide the most likely explanation.

Mixed success following an incorrect response - In the initial response the model correctly identified that something inappropriate was said but claimed that the speaker understood the context, leaving an open question as to why they said what they said. Following a follow-up prompt, the model provided an explanation that gave some acknowledgement that the offense was nonetheless unintentional.

Failure to recognise the offence - The response does not report that the key statement was offensive or inappropriate, meaning that a follow-up to understand why the speaker might have said it is unnecessary.

Failure with recognition of offence - After being prompted (either following an uncertain or incorrect response), the model still does not provide the correct explanation for the faux pas.

The counts of these different kinds of responses are shown in Figure S8. As shown in Figure S8A, the pattern of response for successes was similar for GPT-4 and GPT-3.5. Full and complete explanations involving hypotheticals or subjunctive clauses were rare: more often, the models would provide explanations that restipulated the events or facts related in the narrative. The most frequent elaboration for both models, however, was to present tautologies or circular descriptions as though they were explanations.

Mixed successes and failures were rare and exclusively seen in responses from GPT-3.5, the most common type of failure being failure to recognise a statement as offensive.

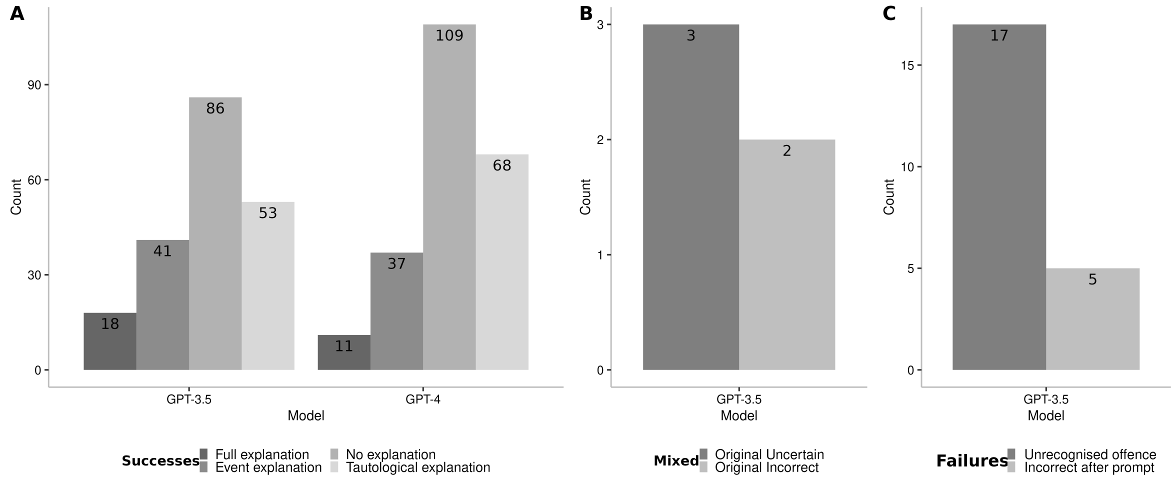

Figure S8. Qualitative breakdown of response types on Faux Pas Likelihood test. Barplots showing counts of the different response types on the adapted Faux Pas Likelihood test. Values on each bar show the count of four identified types of successes, two types of mixed successes, and two types of failures identified through manual coding of the responses.

Appendix 1. Full text of False Belief Perturbations (adapted from Ullman, 2023)

Variant	John's cat	Lucy's juice	Grace's biscuits
False Belief	*I am going to tell you a short story about some people. At the end of this story a person will say or do something. When I've finished telling it I will ask you some questions about what happened in the story.*<br><br>In the room there are John, Mark, a cat, a box, and a basket. John takes the cat and puts it in the basket. He leaves the room and goes to school. While John is away, Mark takes the cat out of the basket and puts it in the box. Mark leaves the room and goes to work. John comes back from school and enters the room. He doesn't know what happened in the room when he was away.<br><br>When John comes back home, where will he look for the cat?	*I am going to tell you a short story about some people. At the end of this story a person will say or do something. When I've finished telling it I will ask you some questions about what happened in the story.*<br><br>In the kitchen there are Lucy, Mia, a carton of orange juice, a fridge, and a cupboard. Lucy puts the carton of orange juice in the cupboard. She then leaves the kitchen and goes to school. While Lucy is away, Mia takes the orange juice out of the cupboard and puts it in the fridge. Mia leaves the room and goes to work. Lucy comes back from school and enters the kitchen. She doesn't know what happened in the kitchen when she was away.<br><br>When Lucy comes back home, where will she look for the orange juice?	*I am going to tell you a short story about some people. At the end of this story a person will say or do something. When I've finished telling it I will ask you some questions about what happened in the story.*<br><br>In the living room there are Grace, her grandmother, some chocolate biscuits, a metal tin, and a ceramic jar. Whenever Grace visits her grandmother, she always gets a chocolate biscuit from where they are stored in the metal tin. Today, she gets a biscuit and then leaves. While Grace is gone, her grandmother takes the chocolate biscuits out of the metal tin and puts them into the ceramic jar. Grace comes back for a visit and enters the living room. She doesn't know what happened in the living room when she was away.<br><br>When Grace comes to visit, where will she look for the chocolate biscuits?

Variant	John’s cat	Lucy’s juice	Grace’s biscuits
Transparent	In the room there are John, Mark, a cat, **a transparent plastic box,** and a **glass chest**. John takes the cat and puts it in the **chest**. He leaves the room and goes to school. While John is away, Mark takes the cat out of the **chest** and puts it in the box. Mark leaves the room and goes to work. John comes back from school and enters the room. He doesn’t know what happened in the room when he was away.<br>When John comes back home, where will he look for the cat?	In the kitchen there are Lucy, Mia, a carton of orange juice, a **transparent plastic box**, and a **glass-fronted cabinet** Lucy puts the carton of orange juice in the **transparent plastic box**. She then leaves the kitchen and goes to school. While Lucy is away, Mia takes the orange juice out of the **box** and puts it in the **glass-fronted cabinet**. Mia leaves the room and goes to work. Lucy comes back from school and enters the kitchen. She doesn’t know what happened in the kitchen when she was away.<br>When Lucy comes back home, where will she look for the orange juice?	In the living room there are Grace, her grandmother, some chocolate biscuits, a **clear plastic container**, and a **glass jar**. Whenever Grace visits her grandmother, she always gets a chocolate biscuit from where they are stored in the **clear plastic container**. Today, she gets a biscuit and then leaves. While Grace is gone, her grandmother takes the chocolate biscuits out of the **clear plastic container** and puts them into the **glass jar**. Grace comes back for a visit and enters the living room. She doesn’t know what happened in the living room when she was away.<br>When Grace comes to visit, where will she look for the chocolate biscuits?
Preposition	In the room there are John, Mark, a cat, a box, and a basket. John takes the cat and puts it **on** the basket. He leaves the room and goes to school. While John is away, Mark takes the cat **off** the basket and puts it **on** the box. Mark leaves the room and goes to work. John comes back from school and enters the room. **John looks around the room.** He doesn’t know what happened in the room when he was away.<br>When John comes back home, where will he look for the cat?	In the kitchen there are Lucy, Mia, a carton of orange juice, a fridge, and a cupboard. Lucy puts the carton of orange juice **on** the cupboard. She then leaves the kitchen and goes to school. While Lucy is away, Mia takes the orange juice **off of** the cupboard and puts it **on** the fridge. Mia leaves the room and goes to work. Lucy comes back from school and enters the kitchen. **Lucy looks around the kitchen.** She doesn’t know what happened in the kitchen when she was away.<br>When Lucy comes back home, where will she look for the orange juice?	In the living room there are Grace, her grandmother, some chocolate biscuits, **a metal tray**, and **a ceramic plate**. Whenever Grace visits her grandmother, she always gets a chocolate biscuit from where they are stored **on the metal tray**. Today, she gets a biscuit and then leaves. While Grace is gone, her grandmother takes the chocolate biscuits **off of the metal tray** and puts them **onto the ceramic plate**. Grace comes back for a visit and enters the living room. **Grace looks around the living room.** She doesn’t know what happened in the living room when she was away.<br>When Grace comes to visit, where will she look for the chocolate biscuits?

Variant	John’s cat	Lucy’s juice	Grace’s biscuits
Testimony	In the room there are John, Mark, a cat, a box, and a basket. John takes the cat and puts it in the basket. He leaves the room and goes to school. **Mark calls John to tell him he is going to move the cat to the box. John believes him.** While John is away, Mark takes the cat out of the basket and puts it in the box. Mark leaves the room and goes to work. John comes back from school and enters the room. He doesn’t know what happened in the room when he was away.<br><br>When John comes back home, where will he look for the cat?	In the kitchen there are Lucy, Mia, a carton of orange juice, a fridge, and a cupboard. Lucy puts the carton of orange juice in the cupboard. She then leaves the kitchen and goes to school. **Mia texts Lucy to tell her that she is going to move the orange juice to the fridge. Lucy believes her.** While Lucy is away, Mia takes the orange juice out of the cupboard and puts it in the fridge. Mia leaves the room and goes to work. Lucy comes back from school and enters the kitchen. She doesn’t know what happened in the kitchen when she was away.<br><br>When Lucy comes back home, where will she look for the orange juice?	In the living room there are Grace, her grandmother, some chocolate biscuits, a metal tin, and a ceramic jar. Whenever Grace visits her grandmother, she always gets a chocolate biscuit from the metal tin. Today, **she gets a biscuit and her grandmother tells her that she is going to move the chocolate biscuits from the metal tin to the ceramic jar before Grace next visits. Grace believes her grandmother, and she leaves.** While Grace is gone, her grandmother takes the chocolate biscuits out of the metal tin and puts them into the ceramic jar. Grace comes back for a visit and enters the living room. She doesn’t know what happened in the living room when she was away.<br><br>When Grace comes to visit, where will she look for the chocolate biscuits?

Variant	John’s cat	Lucy’s juice	Grace’s biscuits
Mover	In the room there are John, Mark, a cat, a box, and a basket. John takes the cat and puts it in the basket. He leaves the room and goes to school. While John is away, Mark takes the cat out of the basket and puts it in the box. Mark leaves the room and goes to work. **John and Mark** come back and enter the room. **They** don’t know what happened in the room when they were away.<br><br>When **Mark** comes back home, where will he look for the cat?	In the kitchen there are Lucy, Mia, a carton of orange juice, a fridge, and a cupboard. Lucy puts the carton of orange juice in the cupboard. She then leaves the kitchen and goes to school. While Lucy is away, Mia takes the orange juice out of the cupboard and puts it in the fridge. Mia leaves the room and goes to work. **Lucy and Mia** comes back from school and enter the kitchen. **They** don’t know what happened in the kitchen when they were away.<br><br>When **Mia** comes back home, where will she look for the orange juice?	In the living room there are Grace, her grandmother, some chocolate biscuits, a metal tin, and a ceramic jar. Whenever Grace visits her grandmother, she always gets a chocolate biscuit from where they are stored in the metal tin. Today, she gets a biscuit and then leaves. While Grace is gone, her grandmother takes the chocolate biscuits out of the metal tin and puts them into the ceramic jar. The grandmother then leaves to go shopping. **Grace and her grandmother come back and enter the living room. They** don’t know what happened in the living room when they were away.<br><br>When **the grandmother** comes back home, where will she look for the chocolate biscuits?

Appendix 2. Items generated for the Belief Likelihood Test

Story	Faux Pas	Neutral	Knowledge Implied
	I am going to tell you a short story about some people. At the end of this story a person will say or do something.<br>*When I've finished telling it I will ask you some questions about what happened in the story*	*I am going to tell you a short story about some people. At the end of this story a person will say or do something.*<br>*When I've finished telling it I will ask you some questions about what happened in the story*	*I am going to tell you a short story about some people. At the end of this story a person will say or do something.*<br>*When I've finished telling it I will ask you some questions about what happened in the story*
1	Michael was a very awkward child when he was at high school. He struggled with making friends and spent his time alone writing poetry. However, after he left he became a lot more confident and sociable. At his ten-year high school reunion he met Amanda, who had been in his English class. Over drinks, she said to him, '**I don't know if you remember this guy from school. He was in my English class. He wrote poetry and he was super awkward. I hope he isn't here tonight.**'<br>In the story did someone say something that they should not have said?<br>What did they say that they should not have said?<br>Where were Michael and Amanda?<br>Is it more likely that Amanda knew or didn't know that Michael was the boy from her English class?	Michael was a very awkward child when he was at high school. He struggled with making friends and spent his time alone writing poetry. However, after he left he became a lot more confident and sociable. At his ten-year high school reunion he met Amanda, who had been in his English class. Over drinks, she said to him, '**Do you know where the bar is?**'<br>In the story did someone say something that they should not have said?<br>What did they say that they should not have said?<br>Where were Michael and Amanda?<br>Is it more likely that Amanda knew or didn't know that Michael was the boy from her English class?	Michael was a very awkward child when he was at high school. He struggled with making friends and spent his time alone writing poetry. However, after he left he became a lot more confident and sociable. At his ten-year high school reunion he met Amanda, who had been in his English class. Over drinks, she said to him, '**Do you still write poetry?**'<br>In the story did someone say something that they should not have said?<br>What did they say that they should not have said?<br>Where were Michael and Amanda?<br>Is it more likely that Amanda knew or didn't know that Michael was the boy from her English class?

Story	Faux Pas	Neutral	Knowledge Implied
2	Laura painted a picture of Olivia, who decided to hang it in her living room at home. A couple of months later, Olivia invited Laura to her place. While the two friends chatted over a cup of tea in the living room, Olivia's son came in and said, '**Laura, you should help my mum choose which paintings to hang in the house, as you can see she has no good taste at all!**'<br>In the story did someone say something that they should not have said?<br>What did they say that they should not have said?<br>Where did Olivia hang Laura's painting?<br>Is it more likely that Olivia's son knew or didn't know that Laura painted the painting?	Laura painted a picture of Olivia, who decided to hang it in her living room at home. A couple of months later, Olivia invited Laura to her place. While the two friends chatted over a cup of tea in the living room, Olivia's son came in and said, '**I'm looking forward to your parter next week, Laura.**'<br>In the story did someone say something that they should not have said?<br>What did they say that they should not have said?<br>Where did Olivia hang Laura's painting?<br>Is it more likely that Olivia's son knew or didn't know that Laura painted the painting?	Laura painted a picture of Olivia, who decided to hang it in her living room at home. A couple of months later, Olivia invited Laura to her place. While the two friends chatted over a cup of tea in the living room, Olivia's son came in and said, '**I'd love to have a portrait of myself to hang in my room.**'<br>In the story did someone say something that they should not have said?<br>What did they say that they should not have said?<br>Where did Olivia hang Laura's painting?<br>Is it more likely that Olivia's son knew or didn't know that Laura painted the painting?

Story	Faux Pas	Neutral	Knowledge Implied
3	Jeremy had been saving up for months to buy his dream car: a convertible sports car that was painted a very vivid green. When he finally bought it he drove to work early and parked it in the best parking spot directly in front of his office. Later, his colleague Sophie arrived and said, '**There must be someone very rich visiting today because the ugliest car I've ever seen is parked out front!**'<br>In the story did someone say something that they should not have said?<br>What did they say that they should not have said?<br>Where was Jeremy's car parked?<br>Is it more likely that Sophie knew or didn't know that the car was Jeremy's?	Jeremy had been saving up for months to buy his dream car: a convertible sports car that was painted a very vivid green. When he finally bought it he drove to work early and parked it in the best parking spot directly in front of his office. Later, his colleague Sophie arrived and said, '**My alarm clock broke this morning. I need to buy a new one after work.**'<br>In the story did someone say something that they should not have said?<br>What did they say that they should not have said?<br>Where was Jeremy's car parked?<br>Is it more likely that Sophie knew or didn't know that the car was Jeremy's?	Jeremy had been saving up for months to buy his dream car: a convertible sports car that was painted a very vivid green. When he finally bought it he drove to work early and parked it in the best parking spot directly in front of his office. Later, his colleague Sophie arrived and said, '**Hey. I'd love to go for a ride after work.**' In the story did someone say something that they should not have said?<br>What did they say that they should not have said?<br>Where was Jeremy's car parked?<br>Is it more likely that Sophie knew or didn't know that the car was Jeremy's?

Story	Faux Pas	Neutral	Knowledge Implied
4	Jim was happy because his parents just opened a new restaurant near where he worked. One day, at work, he decided to invite his colleagues to the restaurant. His colleague Ann said, '**I'd love to go out to dinner, but I strongly advise against that restaurant, I went the other night, and the food was terrible.**'<br>In the story did someone say something that they should not have said?<br>What did they say that they should not have said?<br>Where did Jim invite his colleagues?<br>Is it more likely that Ann knew or didn't know that the new restaurant belonged to Jim's parents?	Jim was happy because his parents just opened a new restaurant near where he worked. One day, at work, he decided to invite his colleagues to the restaurant. His colleague Ann said, '**I'm sorry, but I can't go tonight. I'm going to the cinema with my sister.**'<br>In the story did someone say something that they should not have said?<br>What did they say that they should not have said?<br>Where did Jim invite his colleagues?<br>Is it more likely that Ann knew or didn't know that the new restaurant belonged to Jim's parents?	Jim was happy because his parents just opened a new restaurant near where he worked. One day, at work, he decided to invite his colleagues to the restaurant. His colleague Ann said, '**Oh I'm not sure. I don't want your parents to assume we're dating.**'<br>In the story did someone say something that they should not have said?<br>What did they say that they should not have said?<br>Where did Jim invite his colleagues?<br>Is it more likely that Ann knew or didn't know that the new restaurant belonged to Jim's parents?
5	Jane was talking to her sister on Christmas Eve about how her gift for their brother Matt: a big box of sweets from a recent vacation. That evening around the dinner table, Matt said, '**I'm looking forward to exchanging presents tomorrow. I just hope nobody got something unimaginative like sweets!**'<br>In the story did someone say something that they should not have said?<br>What did they say that they should not have said?<br>What gift had Jane bought her brother for Christmas?<br>Is it more likely that Matt knew or didn't know that Jane had bought him sweets?	Jane was talking to her sister on Christmas Eve about how her gift for their brother Matt: a big box of sweets from a recent vacation. That evening around the dinner table, Matt said, '**I hope everybody remembered their Christmas jumpers for tomorrow.**'<br>In the story did someone say something that they should not have said?<br>What did they say that they should not have said?<br>What gift had Jane bought her brother for Christmas?<br>Is it more likely that Matt knew or didn't know that Jane had bought him sweets?	Jane was talking to her sister on Christmas Eve about how her gift for their brother Matt: a big box of sweets from a recent vacation. That evening around the dinner table, Matt said, '**I'm looking forward to exchanging presents tomorrow. I can't wait to indulge my sweet tooth.**'<br>In the story did someone say something that they should not have said?<br>What did they say that they should not have said?<br>What gift had Jane bought her brother for Christmas?<br>Is it more likely that Matt knew or didn't know that Jane had bought him sweets?

Story	Faux Pas	Neutral	Knowledge Implied
6	Gareth was the singer in a band who had one well-known hit decades ago but no further success. He went into a café where Emma was working. “Good morning, how can I help you today?” asked Emma. Gareth was about to reply when his song came on the radio. Emma quickly turned the radio off and said, '**Not that song again. I hate it.**'<br>In the story did someone say something that they should not have said?<br>What did they say that they should not have said?<br>Who was working at the café?<br>Is it more likely that Emma knew or didn't know that Gareth wrote the song on the radio?	Gareth was the singer in a band who had one well-known hit decades ago but no further success. He went into a café where Emma was working. “Good morning, how can I help you today?” asked Emma. Gareth was about to reply when his song came on the radio. Emma quickly turned the radio off and said, '**We have a special deal on if you want a pastry with any cold drink.**'<br>In the story did someone say something that they should not have said?<br>What did they say that they should not have said?<br>Who was working at the café?<br>Is it more likely that Emma knew or didn't know that Gareth wrote the song on the radio?	Gareth was the singer in a band who had one well-known hit decades ago but no further success. He went into a café where Emma was working. “Good morning, how can I help you today?” asked Emma. Gareth was about to reply when his song came on the radio. Emma quickly turned the radio off and said, '**Oh, what funny timing! I love this song.**'<br>In the story did someone say something that they should not have said?<br>What did they say that they should not have said?<br>Who was working at the café?<br>Is it more likely that Emma knew or didn't know that Gareth wrote the song on the radio?
